# Emergency endovascular repair of aortoiliac aneurysms in COVID-19 times

**DOI:** 10.1590/1677-5449.200173

**Published:** 2021-07-05

**Authors:** Rafael de Athayde Soares, Marcus Vinícius Martins Cury, Luiz Maurício da Silva, Patrícia Weiber Schettini Figueiredo, Danilo Augusto Pereira Nery da Costa, Camila de Freitas Correa, Nayara de Arruda Cáceres, Roberto Saciloto

**Affiliations:** 1 Hospital do Servidor Público Estadual de São Paulo, Division of Vascular and Endovascular Surgery, São Paulo, SP, Brasil.

**Keywords:** COVID-19, endovascular surgery, aortic surgery, ruptured aortic aneurysms, COVID-19, tratamento endovascular, aneurisma de aorta, cirurgia da aorta

## Abstract

In this paper, we describe a case series of four patients who were admitted with emergencies related to aortic aneurysms over a 3-day period and were treated with endovascular repair. The first patient was an 81-year-old female with a history of abdominal pain and a ruptured aortic aneurysm diagnosed by AngioCT-scan. The second patient was a 63-year-old male with a history of oral digestive bleeding and an AngioCT-scan showing an aortoenteric fistula. The third patient was a 77-year-old female with sudden-onset abdominal pain and ruptured right common iliac aneurysm. The fourth patient presented with abdominal pain and an AngioCT-scan showed aortic rupture. All four patients were discharged with no major complications or surgical mortality. These case series show that despite the Covid-19 pandemic situation, since elective surgeries decreased, vascular emergencies have increased.

## Resumo

Relatamos uma série de casos de quatro pacientes consecutivos, admitidos com emergências relacionadas a aneurismas aortoilíacos em um período de 3 dias e submetidos a tratamento endovascular. A primeira paciente, do sexo feminino, com 81 anos e com histórico de aneurisma da aorta, apresentou dor abdominal iniciada nos últimos 12 dias. O segundo paciente era do sexo masculino, com 63 anos e foi admitido com hematêmese 3 dias antes da admissão, com angiotomografia demonstrando fistula aortoentérica. A terceira paciente, do sexo feminino e com 77 anos, foi admitida com quadro de ruptura de aneurisma da artéria ilíaca comum direita. O quarto paciente consecutivo apresentou dor abdominal iniciada 2 semanas antes da internação e aneurisma roto da aorta. Todos os quatro pacientes apresentaram emergências aortoilíacas e receberam alta sem complicações maiores ou mortalidade cirúrgica. O relato desta série de casos demonstra que, apesar da situação pandêmica da COVID-19, uma vez que as cirurgias eletivas diminuíram, as urgências vasculares aumentaram.

## INTRODUCTION

Since the outbreak of the SARS-Cov-2 infection pandemic, a considerable impact on elective surgeries has been observed, with a fall in the number of elective repairs performed. This is because most patients preferred to postpone elective surgery and also because surgical societies all round the world have recommended postponement of elective surgery.^1^ Given the uncertainties related to COVID‐19, the surgical community acknowledges the need for resource preservation, but undue postponement of surgery to treat progressive diseases would result in another public health crisis.^2^

One of the first reports regarding the Covid-19 outbreak and vascular surgeries was published by Ng et al.,^3^ in whose vascular surgery department the number of aorta-related cases have decreased, since they have tended to postpone patients with abdominal aortic aneurysms who were asymptomatic.

The objectives of this paper are to report a case series of patients with consecutive aortoiliac aneurysm emergencies and their outcomes and to present a review of vascular surgery practice during the Covid-19 infection pandemic.

The Research Ethics Committee approved this study (decision number 4.091.832).

## CASE REPORTS

### Case 1

**Figure 1:**
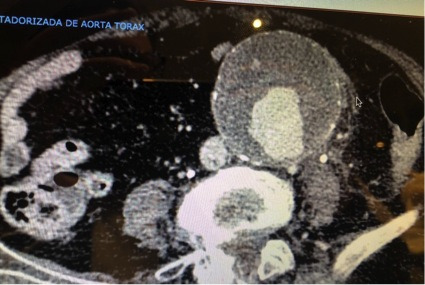
Axial Angio-CT scan showing ruptured aorta in case 1.

**Figure 2:**
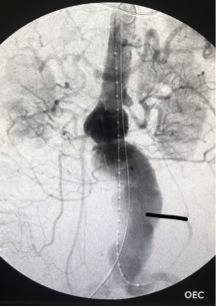
Aortography in anteroposterior plane showing infra-renal aneurysm in case 1.

**Figure 3:**
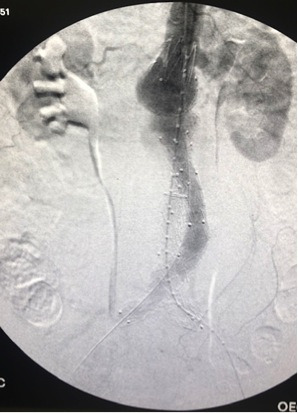
Postoperative anteroposterior aortography with a 36x14x103 Endurant, a 16x20x156 limb extension on the right side and two limb extensions on the left side (16x16x82 and 16x16x124).

An 81 year-old female patient, with arterial hypertension, diabetes, and a history of an aortic aneurysm that had been diagnosed in 2015, presented at dawn on a Saturday and was admitted with abdominal pain with onset during the preceding 12 days. In view of the known history of aortic aneurysm, she underwent an AngioCT-scan that showed a ruptured pararenal aortic aneurysm with maximum diameter of 72mm and retroperitoneal hematoma (Figure 1). Although the aortic neck had conical morphology, it had a length of 20mm before the start of the dilated portion of the aorta (Figure 2). The patient was hemodynamically stable and underwent endovascular aortic repair under general anesthesia, with bilateral groin incisions to expose the femoral arteries. A 36x14x103 Endurant II main body, combined with a 16x20x156 limb extension on the right side and two limb extensions on the left side (16x16x82 and 16x16x124) were successfully implanted (Figure 3). The patient progressed satisfactorily after the surgery and remained hemodynamically stable. On the second postoperative day she had leukocytosis (18 x 10^9^ cells/L) associated with coughing. A chest CT showed left lung consolidation suggestive of bacterial pneumonia. A diagnosis of SARS-Cov-2 was ruled out with laboratory tests and chest CT. She was put on Piperacillin-Tazobactan for 7 days, without needing admission to the intensive care unit and recovered satisfactorily, before being discharged 9 days after the endovascular repair.

### Case 2

**Figure 4:**
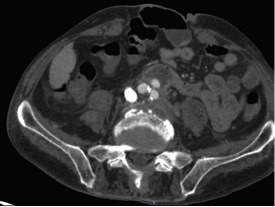
Axial AngioCT-scan showing intraintestinal bleeding, secondary to an aortoenteric fistula in case 2.

**Figure 5:**
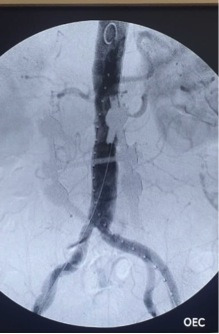
Preoperative angiography in anteroposterior projection.

**Figure 6:**
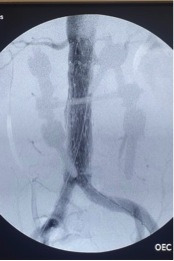
Postoperative angiography in anteroposterior plane with AFX – Endologix 22-70/I16-30 and Vela 25-25/C75.

A 63-year-old male patient with a history of alcoholic liver cirrhosis was admitted during the Saturday morning with a history of oral digestive bleeding with onset 3 days before admission. He underwent upper digestive endoscopy that detected stomach bleeding. His past medical history reveled previous spine surgery, with vertebral fixation due to discitis. Six months later, a huge aortic pseudoaneurysm was diagnosed and an aorto-aortic Dacron bypass had been performed at our hospital. An AngioCT-scan showed an aortoenteric fistula (Figure 4). He was hemodynamically stable and underwent endovascular repair with an Endologix AFX –22-70/I16-30 + Vela 25-25/C75, totally percutaneously, with three Abbott Perclose Proglide devices (Figures 5 and 6). After surgery, the patient progressed well and another upper digestive endoscopy showed no bleeding at all. Additionally, the patient also underwent CT-guided aspiration of a peri-aortic collection which when cultured revealed multisensitive E. coli. Esophagus-stomach-duodenum barium radiography ruled out aorto-enteric fistula. The patient initiated oral diet, without complications, and was discharged on oral antibiotic therapy 11 days after the surgery.

### Case 3

**Figure 7:**
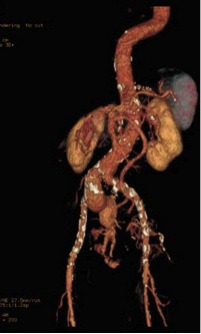
AngioCT-scan in coronal reconstruction with a right common iliac artery rupture in case 3.

**Figure 8:**
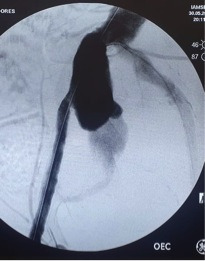
Preoperative aortography in anteroposterior projection in case 3.

**Figure 9:**
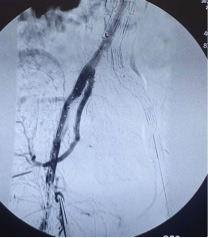
Postoperative aortography in anteroposterior projection in case 3; 24x82 Zenith Cook, 13x74 left limb extension, and 16x39 right limb extension, with a 12x45x58 ZBIS and a 6x100 Viabahn and a 9x80 Fluency in the right internal iliac artery.

The third patient was a 77-year-old female active smoker with hypertension and congestive heart failure who was admitted on the Saturday afternoon with sudden abdominal pain. Once more, an AngioCT-scan showed a ruptured right common iliac aneurysm, without hemorrhagic shock (Figure 7). Under general anesthesia, both femoral arteries were exposed and endovascular repair was carried out with a 24x82 Cook Zenith with a 13x74 left limb extension and 12x45x58 ZBIS on the right with a 16x39 limb extension. There was hypogastric aneurysm involvement, so the device was connected to the upper gluteal artery with a 6x100 Gore Viabahn and 9x80 Bard Fluency (Figures 8 and 9). The patient’s postoperative course was satisfactory and she was discharged 5 days after surgery.

### Case 4

**Figure 10:**
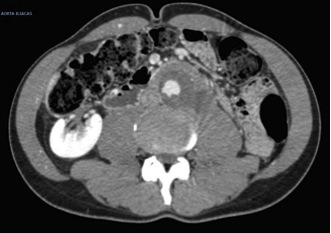
AngioCT-scan in axial plane showing aortic rupture in case 4.

**Figure 11:**
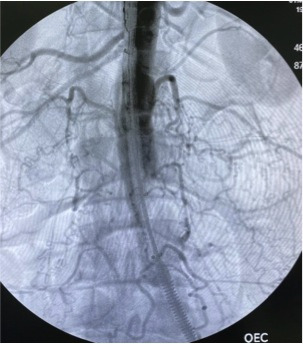
Intraoperative anteroposterior projection aortography in case 4.

**Figure 12:**
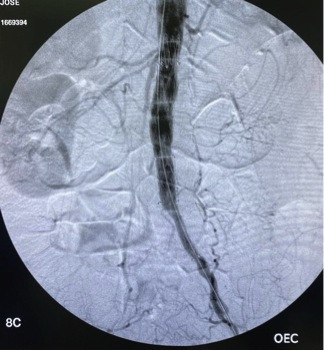
Post-endoprosthesis implant aortography in anteroposterior projection showing 23x14x102 monoiliac Endurant II for the left limb with a 16x10x82 left iliac limb extension.

The fourth consecutive patient was a 64-year-old male active smoker with preexisting right limb claudication who arrived on the Monday afternoon with an abdominal pain that had had onset 2 weeks before admission, with symptoms worsening 2 days before he presented. An AngioCT-scan showed a ruptured aortic aneurysm with 53mm maximum diameter (Figure 10), with right common iliac and external iliac occlusion, and a proximal neck length of 25mm. This patient underwent endovascular repair with a 23x14x102 monoiliac Endurant II for the left limb and a 16x10x82 left iliac limb extension, after left common iliac angioplasty with an 8x40 catheter balloon in order to enable the endograft to pass. A left groin incision was made to expose the femoral artery and a left brachial puncture was used to perform arteriographic control (Figures 11, 12). This patient recovered without further complications and was discharged from hospital 3 days after surgery.

## DISCUSSION

Abdallah et al.,^4^ reported the impact of the Covid-19 pandemic on vascular surgery in Paris. They observed a rising number of acute arterial events in COVID-19 patients with no prior vascular history. These acute arterial events included acute thrombosis of the abdominal aorta, carotid, and peripheral arteries that could be the revealing symptom of COVID-19. The four cases reported in this paper resume very well this condition. This was a very unusual situation in our vascular department; to perform 4 consecutive emergency endovascular aneurysm repairs over the course of 3 days. Even more so if compared with the same period the previous year, May 2019, when there were no cases of emergency aortic repair, only elective repairs. All of these patients had postponed their treatment because of fear of going to hospital and facing the risk of Covid-19 infection, which resulted in them being operated on an emergency basis, which could have ended in a fatal outcome. Fortunately, all four procedures went well and all of the patients survived and were discharged from hospital, without being infected with Covid-19, at least in its symptomatic form, since routine testing of asymptomatic patients had not been included in the infectious diseases protocol of our hospital.

Mousa and Broce^5^ also published a paper reporting the impact of Covid-19 on the vascular interventionist, showing that the number of surgeries was restricted to only urgent or emergency cases, disrupting routine patient flow, causing delays in data entry for new and follow-up visits, and having an unprecedented impact on the vascular specialty. Similarly, in our vascular department elective surgeries had been canceled, due to the need to maintain the intensive care unit prepared to receive patients with Covid-19 infections. All elective aortic aneurysm surgeries were postponed. Only urgent and emergency surgeries are being performed.

Another study by Pini et al.,^6^ observed an increase in the number of urgent aortic aneurysm cases in 2020 compared with 2019 (24% vs. 18%). These results are similar to those we present in this paper, in which we report four consecutive emergency aortic aneurysms over a period of 3 days, and an increasing of urgent aortic aneurysm cases in 2020 compared with 2019 (56% vs 23%). These conditions could be because of the reduction in outpatient visit flows at our department and also the patients’ fear of acquiring Covid-19 infection, situations that can delay appropriate treatment of the patient.

This paper reports four cases of emergency aortic aneurysms that were all treated with EVAR and all of the patients had satisfactory outcomes. The EVAR‐1, DREAM, and OVER trials all showed an early survival benefit for EVAR, when compared to open aortic surgery.^7^^-^9 Large-scale studies, meta-analyses from IMPROVE have shown EVAR to confer reduction in hospital mortality and morbidity in patients with favorable aneurysm morphology.^10^^,^^11^ Therefore, the current best evidence favors an “EVAR first” policy for ruptured AAA, which has been followed in our surgical department with favorable results. Moreover, particularly during the COVID-19 pandemic crisis, the choice of endovascular treatment to manage these aortic emergencies may be preferred to avoid infection during surgery, since contamination of the operating field and personnel may occur because of aerosolization during laparotomy or at the time of evacuation of abdominal gas and smoke during laparoscopy.^12^^,^^13^ A paper published by Safari et al.^14^ analyzing multiple clinical specimens obtained during emergency abdominal surgery in 4 COVID-19 patients showed that SARS-CoV-2 RNA was found in the feces of 3 patients and in the duodenal wall of a patient with perforated peptic ulcer, although real time reverse transcriptase polymerase chain reaction (RT-PCR) assay of abdominal fluid was negative for the virus.

Another interesting finding in this case report was the endovascular repair in a patient with aortoenteric fistula (AEF). Endovascular repair of AEF has emerged as an evolving treatment option with potentially improved mortality outcomes. The patient in this present paper probably had a secondary AEF. A recent meta-analysis reported an in-hospital mortality rate of 7% for 98 well-documented cases of endovascular AEF repair from the literature. When suitable, endovascular treatment of AEF is a safe procedure, with fewer complications and better outcomes than open surgery repair, particularly during the COVID-19 pandemic crisis.^15^

We are facing a pandemic crisis situation that is changing vascular surgery and other surgical specialties, increasing emergency procedures and challenging vascular surgery all over the world. That is the importance of reporting the changing surgical flow and numbers of surgeries, as well as the causes of 4 consecutive ruptured aortic aneurysms in 3 days, and highlighting the necessity of choosing the endovascular approach to avoid COVID-19 infection and contamination.

## CONCLUSION

The Covid-19 pandemic crisis is a challenging situation that has increased the number of urgent and emergency surgeries in the vascular world. The four patients reported in this paper had excellent outcomes with EVAR treatment, showing that vascular surgery departments must be prepared to promptly treat their patients’ urgent needs despite the SARS-Cov-2 situation. Furthermore, compared with the same period last year, May 2019, when there were only elective repairs and no cases of emergency aortic repair in our vascular department, this paper shows a significant increase in aortic emergencies during the COVID-19 pandemic crisis.
